# The Bell’s test: a quantitative test evaluation of inattention and visuospatial neglect in the elderly

**DOI:** 10.1590/1980-5764-DN-2024-0269

**Published:** 2025-09-22

**Authors:** Paulo Roberto de Brito-Marques, Abérico Albanês Oliveira-Bernardo

**Affiliations:** 1 Universidade de Pernambuco, Faculdade de Ciências Médicas, Unidade de Neurologia Cognitiva e Comportamental, Recife PE, Brazil.; 2 Universidade de Perambuco, Hospital Universitário Oswaldo Cruz, Recife PE, Brazil.; 3 Universidade de Pernambuco, Faculdade de Ciências Médicas, Pós-Graduação em Especialização em Neurologia Cognitiva e Comportamental, Recife PE, Brazil.

**Keywords:** Neuropsychological Tests, Spatial processing, Alzheimer disease, Dementia, Attention, Testes Neuropsicológicos, Processamento Espacial, Doença de Alzheimer, Demência, Atenção

## Abstract

Bell’s test, a cancellation task, permits a quantitative and qualitative evaluation of inattention and visual neglect. It was included in a neuropsychological assessment by Gauthier, Dehaut, and Joanette in 1989. It is a quick test that can detect visuospatial changes.

**Objective:**

To demonstrate the importance of Bell’s test in elderly individuals without visuospatial complaints. To evaluate Bell’s test in different levels of schooling, age groups, Mini-Mental State Examination (MMSE), and the Modified Mini-Mental State (MMMS) tests.

**Methods:**

A cross-sectional, randomized study was carried out on 278 elderly people, aged between 60 and 89 years old, with a mean age of 69.4 (±6.8 years standard deviation — SD). Among the participants, 73.9% of the females lived in Olinda City, Brazil. Age was stratified every five years between 60 and 89 years old, and schooling levels were categorized into four subgroups, ranging from illiterate to more than eight years old. Each participant underwent an analysis of age, sex, education, risk factors, MMSE, the modified MMMS, and Bell’s test.

**Results:**

The correlation between the A and B errors and age was statistically significant; as age increases, the number of errors also increases. A near significant and strong correlation was observed in individuals aged above 84 and between 60 and 64. Correlation between MMSE, MMSM, and Bell’s test showed a significant, moderate negative correlation.

**Conclusion:**

Increasing age worsens the results of the Bell test. The MMSE and the MMMS tests showed a direct relationship with the results of the Bell test.

## RESUMO.

O teste de Bell, um dos testes de cancelamento, permite fazer uma avaliação quantitativa e qualitativa da inatenção e da negligência visual. Esse teste foi descrito à avaliação neuropsicológica por Gauthier, Dehaut e Joanette (1989). É um teste rápido que pode detectar alterações visuoespaciais.

**Objetivo:**

Demonstrar a importância do teste de Bells em idosos sem queixas visoespaciais. Avaliar o teste de Bell nos diferentes graus de escolaridade, idade, Miniexame do Estado Mental (MEEM) e Miniexame do Estado Mental Modificado (MEMM).

**Métodos:**

Um estudo de corte transversal e randomizado foi realizado em idosos na cidade de Olinda, Brasil. A população consistia em 278 idosos com idades entre 60 e 89 anos, e média de idade de 69.4 (desvio padrão — DP±6.8 anos), dos quais 73.9% eram do sexo feminino. Os participantes foram estratificados pela idade em seis subgrupos entre 60 e 89 anos. A escolaridade foi dividida em analfabetos, um a quatro anos, cinco a oito anos, e mais de oito anos. Todos foram submetidos a avaliação por idade, escolaridade, MEEM, MEMM, comparados com o teste de Bell.

**Resultados:**

Houve correlação estatística entre os erros do grupo A e do grupo B com relação à idade. Para idade maior de 84 anos e entre 60 e 64 anos a correlação foi quase significativa e forte. A correlação entre MEEM, MEMM e o teste de Bell mostrou coeficiente negativo e moderada significância estatística.

**Conclusão:**

Com o aumento da idade, pioram os resultados. Os testes de MEEM e do MEMM mostraram relação direta com os resultados do teste de Bell.

## INTRODUCTION

 The Bell test is a cancellation test widely used in neuropsychological assessment to examine visuospatial functions, selective and concentrated attention, speed, and psychomotor coordination^1^. The cancellation test was developed by Deny-Brow (1963) when studying monkeys with cortico-mesencephalic fiber lesions, obtaining various visuospatial alterations^2^. The application of the cancellation test in the clinic was introduced ten years later in neuropsychological assessment by Albert, addressing cases of spatial neglect disorder in patients with brain damage^3^. 

 The Bell test is a sensitive test for assessing visual neglect^4^, including patients with traumatic brain injury^5^. It can be used for people with little education and for those who have had an education. However, it must be differentiated when explaining the test to individuals with and without education, including those with intellectual disabilities^6^. When performed, it allows visualization of the omitted targets and their quantification in individuals with autonomy and independence, and a reduction in the quantification of omitted targets indicates clinical improvement^4^. Because the Bell test is more sensitive to visuospatial attention in the right cerebral hemisphere (RCH), it can detect attention deficits in functionally independent elderly individuals. The Bell test detects mild to moderate visual neglect, besides visual attention deficit^4^. It can be performed using two versions; canceling the bells by marking them with circles requires more time than canceling them with a dash^7^, which could alter the performance time. An error in canceling the target by confusing it with another target may not occur only due to visual neglect, but also due to hemianopsia or using the non-dominant hand.^8^

 The Bell test does not have a single cutoff point for cases of visual neglect; it generally varies according to age and education.^9^ However, half of the control group made no omission, leading to the recommendation that if a subject presents a 3-point deficit, on one or another side of the page, it is considered to be a lateralized attention deficit, if he or she omits six bells or more in the contralateral half of the test, for example in the RCH, he or she should be suspected of having visual neglect, which will probably correspond to left visual neglect in most cases.^4^ The visual perception of the RCH is present in both visual fields, while that of the left cerebral hemisphere is in the right field, the latter being less severe.^10^ Comparing Bell and the classic Albert tests, the former proved more sensitive in assessing spatial attention^11^. Right visual negligence detected by Bell’s test, a subcomponent of the same domain, may raise suspicion of mild Alzheimer’s disease^12^. Both versions of the Bell test have been validated for the Brazilian population^13^. Therefore, the objective was to quantify the Bell test score in elderly individuals on their age and education levels. 

## METHODS

### Study design

 A cross-sectional, randomized study was conducted in an elderly population living in a community in the city of Olinda, state of Pernambuco, Brazil. 

 The sample, containing 278 individuals, was based on the following data:

- The neighborhood’s streets were listed and drawn, using an updated map.
- On each street, all participants aged between 60 and 90 were tested, complying with the research inclusion criteria.

The sample size was calculated based on a pilot observation, a population standard deviation (SD) of 3.8, a confidence interval of 95%, and a precision of 0.5, obtaining 278 participants, adding a margin of 3%, to prevent a possible drop-out. The population consisted of 278 elderly people aged between 60 and 89 years, and the average age was 69.4 (±6.8 years SD), 73.9% were females. Of the total number of the sample, 98.5% were right-handed. Participants were stratified by age into six subgroups: 60–64, 65–69, 70–74, 75–79, 80–84, and 85–89. Schooling was categorized into four subgroups: illiterate, between one and four years, between five and eight years, and more than eight years. All subjects underwent a neuropsychological assessment, including demographic data and the MMSE^14^ and MMMS^15-17^ — the latter test was carried out in Brazil with individuals between 60 to 92 years old—and compared to Bell’s test^4^. The likelihood of mild cognitive impairment and dementia in this elderly population varied based on the degree of aging, habits, risk factors, and culture, not exceeding 10%^18-20^. The study was approved by the local ethics committee. The confidentiality of the research was guaranteed by those responsible for the participants’ records. Exclusion criteria were uncorrected visual and auditory deficits, neurological and psychiatric disorders, or joint disease that hindered motricity, and participants with intellectual disabilities were differentiated from illiterates by not recognizing colors, use of money and how to use a can opener. 

### Material

**Figure 1:**
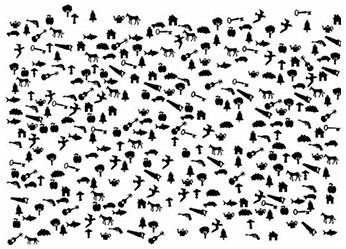
The Bell test (reduced size) task sheet was presented to the subject^4^.

 A 21.5 x 28 cm sheet containing different outlines of objects (house, horse, bell...) is presented to the subject (Figure 1)^4^. A total of 35 targets (bells) are distributed equally on the sheet. All drawings are black like Chinese shadows. 

### Procedure

 The examiner is sitting in front of the patient. First, a demo sheet is presented to the latter. This sheet shows various enlarged figures and a bell, which is circled. The patient is asked to name each figurine pointed to by the examiner’s finger to ensure that the patient correctly recognizes the different objects. In cases where the patient presents language disorders or when the examiner suspects comprehension problems, it is possible for the patient to use cards representing the different figures, and he/she can affix them one by one to their equivalent located on the demonstration sheet to demonstrate understanding. The page with the Bell test was virtually divided into A and B half sides (A and B groups), keeping the projections of the right and left visual fields. The examiner gives the following oral instructions: "Your task is to use this pen to circle all the bells on the sheet of paper that I will place in front of you, without wasting time. You will begin this task when I give you the starting signal: "go ahead", and you will stop when you estimate that you have circled all the bells. I also ask that you avoid moving if possible or tilt your bust." In case of difficulties in understanding, the examiner will demonstrate the task above. The test sheet (A4 size) will be placed in front of the patient, with the black marker at the bottom of the page, in the patient’s mid-sagittal plane. The test sheet will be provided to the patient after receiving all instructions. The participant starts the task and, if he stops before all the bells are circled, the examiner is authorized to issue a warning message like this: "Are you sure all the bells are well surrounded? Please check again"^4^. A qualitative evaluation of the test was not performed. 

### Score and interpretation

 The number of bells circled and the time it took the patient to perform the task were noted. The maximum score is 35. The lack of three bells on the test page indicates inattention. The lack of six or more bells on the right or left side of the page indicates unilateral spatial neglect (USN). Completion time was not a valuable indicator of success or neglect. Normal subjects take from 1 to 5 minutes with no omissions^4^. Sex did not influence Bell’s test to score omission performances. All elderly subjects between 60 and 89 years old completed the Bell Test in 3 to 5 minutes^4^. This work did not define threshold values (percentiles 5 and 95) allowing us to decide on the pathological nature of a patient’s performance. 

### Statistic analysis

 The Statistical Package for the Social Sciences (SPSS) version 20.0 was used to analyze the relationship between errors in Bell’s test compared with variables such as education level, age, MMSE, and MMMS test performance using Pearson’s correlation. Pearson’s correlation assesses the strength and direction of linear associations between two variables, with p-values less than 0.05 considered statistically significant. 

## RESULTS

 This study was carried out with 278 elderly people, 73% of which were females. The correlation between the errors of the A and B groups, stratified by schooling degree and age, showed a negative and very low correlation coefficient, indicating no statistical significance; the educationalgreater than effects were small. There was no evidence of errors in groups A and B according to schooling grade compared to MMSE, respectively. 

 The correlation between the A and B errors according to age, a positive and lower correlation coefficient, and a lower *p*-value are very significant (Table 1). 

**Table 1:** Pearson’s correlation between both errors according to age.

	r	p-value	Regression equation
Errors A	0.28362	<0.001	Y=-6.71733+0.11939*X
Errors B	0.26574	<0.001	Y=-6.11406+0.10778*X

 Subjects over 84 years, and between 60 and 64 years have almost a significant and strong correlation, which means that extreme age B groups greatly influence the errors (Table 2). The other groups showed no relevant statistics. 

**Table 2:** Pearson correlation between the B errors according to age group.

	r	p-value	Regression equation
60–64	-0.26773	0.02022	Y=16.42157–0.25153*X
65–69	0.00927	0.93128	Y=0.11059+0.00958*X
70–74	0.07308	0.59947	Y=-10.85367+0.1756*X
75–79	0.22095	0.22427	Y=-54.6861+0.74439*X
80–84	0.18227	0.49927	Y=-49.65823+0.64557*X
more than 84	-0.64870	0.03083	Y=69.5234–0.7617*X

 A negative and moderate correlation coefficient was observed compared to the MMSE test and a lower *p*-value, which was significant (Table 3). 

**Table 3:** Pearson’s correlation between the errors of groups A and B according to the Mini-Mental State Examination (MMSE) test.

MMSE test	r	p-value	Regression equation
Errors A	-0.46506	<0.001	Y=8.88971–0.32491*X
Errors B	-0.43042	<0.001	Y=7.89549–0.28975*X

 A negative and moderate correlation coefficient compared to the MMMS test and a lower p-value were statistically significant; the score increased as both A and B test errors decreased, and vice-versa (Table 4). 

**Table 4:** Pearson’s correlation between the A and B errors according to the Modified Mini-Mental State test.

	r	p-value	Regression equation
Errors A	-0.526	<0.001	Y=10.40591–0.35558*X
Errors B	-0.46095	<0.001	Y=8.82893–0.30025*X

## DISCUSSION

 In this current study, the sample size calculated was 278 elderly people aged between 60 and 89 years, with an average age of 69.9, and 73% were females. According to Oldifield^21^, of the total samples, 98.5% were right-handed. There was no direct interference from schooling on the Bell test on the largest sample size, maybe a more detailed explanation to illiterate and 1 and 4 groups. In most cases, age and education did not show differences in the results of the Bell test. On the other hand, as seen in cognition studies along with low education, cultural factors can also influence both positive and negative outcomes^15-17^. The comparison of traditional and digital education tasks was more favorable for those with five or more years of schooling^22^. The use of technology is promising, with some reservations for those with lower levels of education, as well as the possibility of cognitive decline^23^, although it has some advantages^24^. We observe that increasing age worsens the result on the Bell test, and both the MMSE^14^ and the MMMS^15-17^ tests were guided in the direction of the outcome of Bell’s test. 

 The type of test can influence the results, potentially affecting the target cancellation. It has been shown that the pseudoneglect phenomenon is more evident in certain tasks, such as line bisection, and probably also in the representation task of France’s map and writing^25^, but was not mentioned in the Bell test. It is believed that the greatest sensitivity of visual attention occurs in the left visual field because the right cerebral hemisphere maintains attention in both visual fields, and the left cerebral hemisphere maintains it in the right field, especially in angular and supramarginal gyri^10,26,27^. However, the Bell test was not mentioned. When this symptom occurs it is of similar severity in acute left brain injury as in patients after acute right brain injury^10^. 

 On each side of the paper, right or left visual field, the omission of three targets indicates visual inattention in that field^4^. Although the Bell test was designed to assess USN, it can also measure unilateral attention failures^11^. Approximately 17% of the adolescents have visuospatial deficits and 4% have USN^28^. About 50% of patients with neglect show ipsilesional target re-exploration in neglect tasks and daily life and also return to previously visited locations that no longer carry a target^29^. Two groups of elderly individuals in our study, in extreme positions, showed visuospatial alterations. The first was over 84 years of age and the second was between 60 and 64 years. The elderly group likely justified their visuospatial failures due to the aging process. On the contrary, the group aged 60 to 64 attributed their ones to an acquired neurodevelopmental disorder that might have caused visual neglect^30^. 

 Education has been one of the obstacles in clinical neuropsychology studies^15-17^. In our study, schooling effects were minimal. However, the A and B groups of elderly individuals presented statistical differences when Bell’s test was compared with the MMSE, with a directly proportional response. No differences were found when comparing groups A and B^15-17^. 

 The comparison between the two groups, A and B of the Bell test, with both the MMSE and MMMS tests showed a significant difference. This result leads us to think about the possibility of comparison tests working as a guide to the results of Bell’s test, positive and negative inverted correlations: if the tests increase, the results of the Bell test decrease, and vice versa. When comparing MMSE x education and MMMS X Bell’s test there is a similar result^14-17^. 

 The main limitation was the lack of studies on the Bell test in the Northeast region of Brazil to compare with our results, including studies with illiterate elderly people and those with low schooling. Future research should develop more studies using different factors such as age, education, MMSE, and MMMS, besides attention and visuospatial organization in the elderly population of Northeast Brazil. 

 In conclusion, the Bell test showed that it can be used in elderly populations between 60 and 89 years old; education does not have a direct significant influence on its results; aging can alter the tone of sustained attention and reaction speed, impairing the perception of older people; the results of Bell’s test are inversely proportional to aging; there is no quantitative difference between the targets in the brain hemispheres. More studies on elderly and longlived individuals should be carried out to provide better clarifications about this age group and Bell’s test. 

## Data Availability

The datasets generated and/or analyzed during the current study are available from the corresponding author upon reasonable request.
